# Digastric Muscle Phenotypes of the Ts65Dn Mouse Model of Down Syndrome

**DOI:** 10.1371/journal.pone.0158008

**Published:** 2016-06-23

**Authors:** Tiffany J. Glass, Nadine P. Connor

**Affiliations:** 1 Department of Surgery, University of Wisconsin, Madison, Wisconsin, United States of America; 2 Department of Communication Sciences and Disorders, University of Wisconsin, Madison, Wisconsin, United States of America; Rutgers University -New Jersey Medical School, UNITED STATES

## Abstract

Down syndrome is frequently associated with complex difficulties in oromotor development, feeding, and swallowing. However, the muscle phenotypes underlying these deficits are unclear. We tested the hypotheses that the Ts65Dn mouse model of DS has significantly altered myosin heavy chain (MyHC) isoform profiles of the muscles involved in feeding and swallowing, as well as reductions in the speed of these movements during behavioral assays. SDS-PAGE, immunofluorescence, and qRT-PCR were used to assess MyHC isoform expression in pertinent muscles, and functional feeding and swallowing performance were quantified through videofluoroscopy and mastication assays. We found that both the anterior digastric (ADG) and posterior digastric (PDG) muscles in 11-day old and 5–6 week old Ts65Dn groups showed significantly lower MyHC 2b protein levels than in age-matched euploid control groups. In videofluoroscopic and videotape assays used to quantify swallowing and mastication performance, 5–6 week old Ts65Dn and euploid controls showed similar swallow rates, inter-swallow intervals, and mastication rates. In analysis of adults, 10–11 week old Ts65Dn mice revealed significantly less MyHC 2b mRNA expression in the posterior digastric, but not the anterior digastric muscle as compared with euploid controls. Analysis of MyHC 2b protein levels across an adult age range (10–53 weeks of age) revealed lower levels of MyHC 2b protein in the PDG of Ts65Dn than in euploids, but similar levels of MyHC 2b in the ADG. Cumulatively, these results indicate biochemical differences in some, but not all, muscles involved in swallowing and jaw movement in Ts65Dn mice that manifest early in post-natal development, and persist into adulthood. These findings suggest potential utility of this model for future investigations of the mechanisms of oromotor difficulties associated with Down syndrome.

## Introduction

Down syndrome (DS) is caused by a trisomy of the 21^st^ chromosome and affects approximately 1 out of 691 live births[1]. Many children with DS show delayed or aberrant development of oral motor functions involving jaw and tongue movement, which can include poor jaw control and difficulty biting^,^[2–4]. These deficits may be associated with developmental delays and impairments of speech and feeding that can affect individuals throughout the lifespan[5–7]. Related challenges that occur with increased frequency in this syndrome can include oropharyngeal dysphagia[8–10]. The mechanisms of impairment underlying oral motor difficulties affecting feeding and swallowing in DS are complex, and can involve anatomical differences and medical comorbidities as well as underlying etiologies that remain unclear.

The Ts65Dn mouse model of DS is currently one of the most comprehensively studied models of DS[11, 12]. It features a partial trisomy of the distal portion of mouse chromosome 16; a region syntenic to the human 21^st^ chromosome[13]. The Ts65Dn model also includes a triplication of a small portion of mouse chromosome 17; involving 60 genes that are not implicated in DS[14]. Although there has been tremendous work in the use of Ts65Dn to identify mechanisms of cognitive impairment in DS[15], and gross motor and muscle phenotypes have been reported in this model[12, 16, 17], relatively less work has been completed to assess Ts65Dn for utility in modeling oromotor or feeding difficulties present in the human population.

Like humans, mice initially derive the majority of their nutrition through nursing, and subsequently develop the capacity to chew and swallow solid food. This oromotor shift involves significant postnatal alteration in oral management of the food bolus[18]. Previous studies of wild-type mice have shown that developmental feeding transitions from nursing to chewing solid food are accompanied by significant shifts of myosin heavy chain (MyHC) isoform profiles of head and neck muscles involved in feeding [19–21]. These MyHC isoform profile changes have been attributed to adaptive transitions in muscle biochemistry imposed by the functional demands of mastication. As the primary component of the muscle fiber thick filament, MyHC is integral in determining muscle contractile properties. Different isoforms of MyHC are associated with distinct muscle contraction characteristics, and therefore the MyHC isoform profile, or composition, of a given muscle is a strong determinant of muscle fiber type[22]. MyHC 2b is associated with rapid contraction speeds, increased force production capability, and increased expression levels in some head and neck muscles during the period of postnatal development that coincides with the post-weaning feeding transition[19, 21, 23].Thus, MyHC isoform profiles provide a sensitive indication of muscle function in specific muscles critical for murine deglutition[24]. In the context of investigations targeting oromotor impairments associated with DS, MyHC isoform profiles can provide information about biochemical hallmarks of muscle development phenotypes that may occur in this syndrome.

Due to the prevalence of feeding challenges encountered by many individuals with DS, we evaluated the Ts65Dn mouse model for phenotypes involving muscles of mastication and deglutition. We tested the hypotheses that Ts65Dn mice have reductions in the speed of oral movements involved in feeding and swallowing as well as reductions in MyHC 2b.

## Materials and Methods

### Mice

This study was performed according to a protocol approved by the University of Wisconsin School of Medicine and Public Health Animal Care and Use Committee (ACUC). Mice were housed in mixed-genotype cages in a University of Wisconsin School of Medicine and Public Health Laboratory Animal Resources Facility. Mice were kept on a 12:12-hr reverse light-dark cycle. A Ts65Dn mouse colony was established from Jackson strain #005252 (B6EiC3Sn.BLiA-Ts(17^16^)65Dn/DnJ). The colony was maintained by breeding trisomic female mice to B6EiC3Sn.BLiAF1/J males. Breeding mice were fed Teklad rodent diet 2019, and at weaning, mice were transitioned to Teklad rodent diet 8604. Pups were weaned at 21 days of age. Tissue for genotyping was obtained either from tail clips after euthanasia in p11 mice, or from ear clips taken at the time of weaning in older mice. Genotyping was accomplished by Mmu17^16^ translocation breakpoint separated PCR [13] or by Transnetyx, Inc (Cordova, TN) using primers to the translocation breakpoint region. Mice for these studies were p11 (11 days old) or young (5–6 weeks old). Additional adult mice across a wide adult age range (10–53 weeks old) were used to examine changes in MyHC isoform profiles with age. Sex was not determined for the p11 age group, and the young and adult age groups were comprised of male mice. Incisors in all mice were observed as normal with normal occlusion. Videofluoroscopic assessments of swallowing function were performed in 27 mice, and mastication rates were quantified in 21 mice. Following behavioral assessments, mice were euthanized by CO_2_ asphyxiation and processed for tissue analysis assays. Workers conducting and analyzing assays were blinded to mouse genotypes through the use of alphanumeric codes for mouse identification and sample identification.

### Muscle selection and isolation

**Fig 1:**
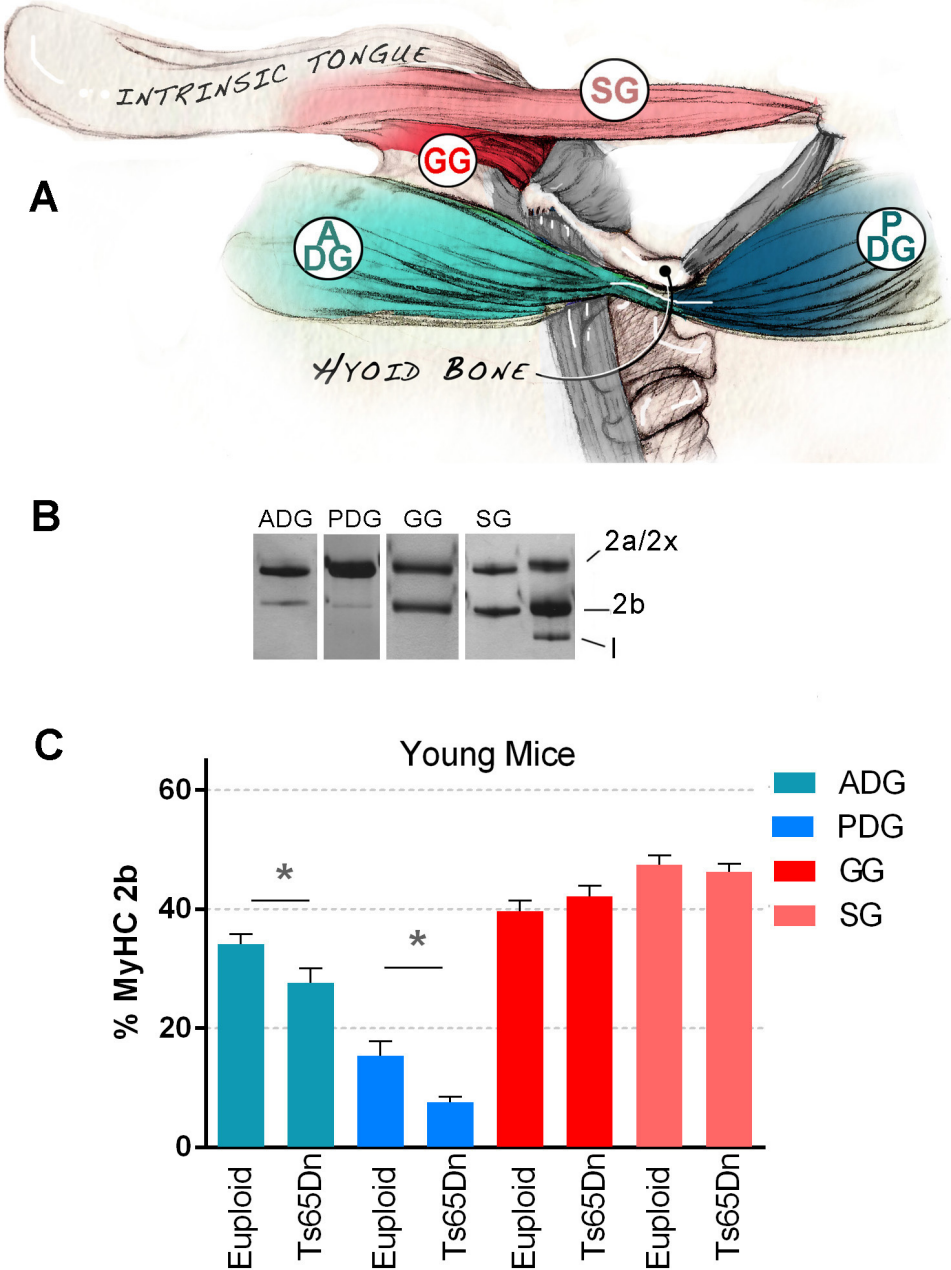
Muscles Selected for Analysis of Young Mice. **A)** Genioglossus (GG), Styloglossus (SG), Anterior belly of the digastric (ADG), and Posterior belly of the digastric (PDG) were selected from young mice for analysis of MyHC isoform composition. **B)** Representative SDS-PAGE gel excerpts. 2a/2x = position of myosin heavy chains 2a and 2x. 2b = myosin heavy chain 2b. I = myosin heavy chain I. **C)** Relative levels of the MyHC 2b isoform protein in each muscle, compared by genotype. Mean and SEM. (* indicates p ≤ .05).

Following euthanasia, a microdissection microscope was used to permit isolation of muscles through a ventral approach in supine mice. Four muscles involved in oral bolus movement and feeding were selected for MyHC phenotype screening to permit empirical identification of optimal candidates for subsequent analysis. These were the genioglossus (GG; involved in tongue protrusion and creation of bolus driving pressures), the styloglossus (SG; involved in tongue retrusion), the anterior belly of the digastric (ADG), and the posterior belly of the digastric (PDG), which are both involved in jaw movement and stabilization of the hyoid bone. Muscles are pictured in Fig 1A.

### SDS-PAGE

GG, SG, ADG, and PDG were removed from euthanized mice and homogenized through mechanical disruption and sonication. Total protein was isolated through previously described methods[25]. A large-format Hoefer SE 600 series sodium doedecyl sulphate polyacrylamide gel electrophoresis (SDS-PAGE) system was used to separate 400 ng of protein samples by molecular weight. Samples were run at 8°C for 29–32 hours though a 6% acrylamide, 30% glycerol separation gel and a 4% acrylamide, 30% glycerol stacking gel[26, 27]. MyHC isoforms were detected though silver staining and identified thorough relative mobility as previously described[28]. Optical density of each band was digitally assessed thorough Un-Scan-It Gel Analysis Software (Silk Scientific, Inc.) and was used to determine MyHC isoform composition of each muscle, calculated as a relative percentage of total MyHC. Proteins from soleus and extensor digitorum longus (EDL) muscles were used as control samples to verify the relative positions of MyHC I and MyHC 2b, respectively, as previously described[28].

### Western Blot

MyHC proteins were transferred from SDS-PAGE gels to PVDF membranes overnight. Following transfer, Ponceau S staining of membranes permitted the locations of protein bands to be indicated. Blots were then blocked in 2.5% milk in TBS, and incubated overnight in primary antibodies to MyHC isoforms; BF-F3 (MyHC 2b), BF-32 (MyHC I and MyHC 2a), and A4.840 (MyHC I), (DSHB, Iowa City, IA) at a concentration of 1:20. Blots were rinsed, incubated in secondary antibodies conjugated to HRP, and developed with DAB.

### qRT-PCR, Microscopy, Microphotograpy, and Image Analysis

Because SDS-PAGE assessments reported below detected significant differences between Ts65Dn and euploid groups in the digastric muscles only, these muscles (ADG, PDG) were chosen for subsequent assays of gene expression and myofiber phenotypes.

#### qRT-PCR

ADG and PDG muscles were isolated from euthanized mice, frozen immediately in liquid nitrogen, and kept at -80°C until use. Each muscle was disrupted using mortar and pestle with liquid nitrogen, followed by further homogenization using Qiagen Qiashredder columns. RNA isolation and DNA removal were achieved with the RNeasy Fibrous Tissue Mini Kit (Qiagen). DNA was additionally removed from samples through treatment with a Turbo DNA-free Kit (Ambion). RNA concentration and purity were determined using a Nanodrop 1000 (Thermo Scientific). cDNA was synthesized per manufacturer instructions using 100 ng RNA per sample, and a High Capacity RNA-to-cDNA kit (Applied Biosystems). qRT-PCR was performed using Taqman Primers (S1 Table) and Taqman Universal Master Mix (Applied Biosystems). qRT-PCR reactions were performed on a 7500 Fast Real-time PCR system (Applied Biosystems). Each biological sample analyzed by qRT-PCR was derived from one muscle from one mouse. For each biological iteration of the experiment, all experimental and control samples were processed in triplicate, for all genes of interest, in the same 96-well plate, alongside RT-negative and no-template controls. Euploid control muscles were set as the biological reference samples. Relative quantitation (RQ) values were determined through the δ-δ-C_t_ method, with expression differences indicated as a fold change relative to the biological reference muscle of the control animal. Both *Bactin* and *Gapdh* were used as the control reference genes in all experiments. C_t_ values indicated no significant differences in expression of the housekeeping genes between muscles or genotype.

#### Immunofluorescence

ADG and PDG muscles were isolated from euthanized mice, embedded in OCT, and frozen in isopentane cooled in liquid nitrogen. Muscles were sectioned at a thickness of 10 microns onto slides, blocked in 1% BSA and .1% Tx-100 in PBS for 10–20 minutes, and stained with primary antibodies BF-F3 (DSHB), and anti-laminin (Sigma) at 4°C overnight. Secondary antibodies (anti-mouse IgGM AF 555 and anti-rabbit AF 488) were applied at concentrations of 1:500 (AF 555) and 1:1000 (AF 488) in 10% normal goat serum in PBS for one hour at room temperature. Nuclei were visualized with DAPI.

#### Microphotography and Image Analysis

Photographs were acquired with a 10X objective using CellSens software, DP73 Olympus camera, and Nikon Eclipse E600 epifluoresence microscope. One entire cross-section from each muscle per sample was photographed and photomontages were manually assembled in Adobe Photoshop CC. Adobe Photoshop CC analysis tools were used to manually count all analyzable myofibers in the section according to positivity for MyHC 2b labeling and the presence of centrally-located nuclei. Cross-sectional area (CSA) was determined using the MATLAB application SMASH[29].

### Mastication Assays

Prior to the start of the assay (18–21 hrs), mice were transferred to individual standard cages and food was withheld. A standard hard mouse food pellet (Harlan Teklad 8604) was offered at the start of the videotaping period. Subsequent mastication was videotaped at 60 fps with the mouse in lateral profile. Immediately following videotaping, food was restored to the mice *ad libitum*. Videos were manually analyzed to quantify mastication rates, using previously described methods[30].

### Videofluoroscopy

A 2:1 ratio of Fritos^®^ Brand Mild Cheddar Flavored Cheese Dip and 40% wt/v barium was used for videofluoroscopic assessment of swallow function. Mice to be assessed through videofluoroscopy were offered the cheese for five days prior to testing. Mice were additionally food regulated overnight prior to the testing session. On the morning of testing, mice in individual cages were offered the barium and cheese mixture on a small platform placed on the cage floor. Subsequent feeding was recorded at a rate of 30 fps with an Artis Zee (Siemens Healthcare, Forchheim, Germany). The videofluoroscopic swallowing study (VFSS) videos were analyzed in Image J using selected measures for high-power VFSS for mice as previously described[31]. Swallow rate was calculated by analyzing no fewer than three 2-second episodes of continuous feeding. The number of consecutive swallows during each 2-second interval were counted and averaged for each mouse[31]. Interswallow Interval (ISI) was assessed by determining the amount of time elapsing between consecutive swallows during continuous feeding, and was averaged from 3 to 5 iterations per mouse[31].

### Behavioral Analysis Reliability Testing

Mastication and VFSS assays were randomly reanalyzed to address the potential for rater error. Intrarater and interrater reliability for quantitative measures were determined through re-analysis of a random selection of no fewer than 10% of video clips and subsequent calculation of intraclass correlation coefficients (ICC). VFSS and mastication intra-rater and inter-rater ICC average measures exceeded .9 for all assays. This indicated sufficient reproducibility of analysis within and across raters.

### Statistical Analysis

Criterion for statistical significance was set at α ≤ .05. SDS-PAGE and immunofluorescence data were analyzed by unpaired t-tests, with Welch’s correction when assumptions for a t-test were not met, or two-way analysis of variance (ANOVA) where appropriate. qRT-PCR data were analyzed by single-sample t-test. For some measures, incidental sample loss resulted in smaller sample sizes and thus smaller degrees of freedom. Statistical analysis was completed using GraphPad Prism 6 (GraphPad Software, Inc.), IBM SPSS version 23 (Armonk, NY: IBM Corp), and SAS statistical software v.9.4 (SAS Institute, Cary, NC).

## Results

### Expression of MyHC 2b isoform protein in select muscles of young Ts65Dn

MyHC isoform composition of each muscle was analyzed by SDS-PAGE (Fig 1B). The relative position of the mouse MyHC 2b isoform in silver-stained gels was confirmed by western blot (S1 Fig). Compared with the euploid control group, the Ts65Dn group showed significantly reduced levels of MyHC 2b in the ADG [t(13) = 2.26, p = .04] and PDG [t(10.20) = 2.91, p = .02] only (Fig 1C). These results permitted empirical prioritization of the ADG and PDG muscles for subsequent interrogation of biochemical muscular differences in Ts65Dn.

### Changes in MyHC2b protein expression in the anterior and posterior digastric muscles during post-natal development

**Fig 2:**
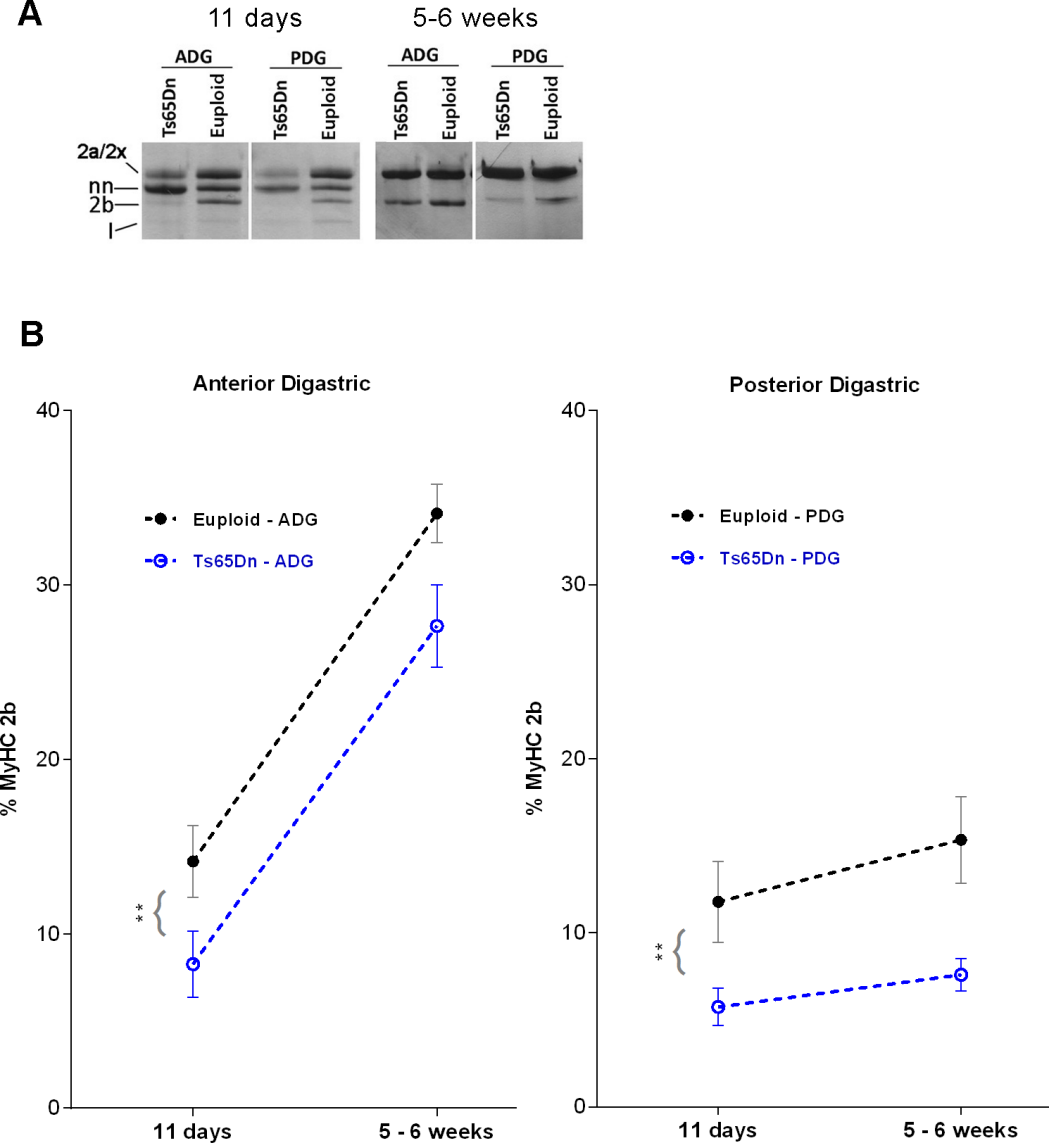
Developmental trajectory of MyHC 2b protein levels in digastric muscles of Ts65Dn assessed by SDS-PAGE. A) SDS-PAGE gels comparing Ts65Dn of different stages of postnatal development to age-matched euploid controls. 2a/2x = position of myosin heavy chains 2a and 2x. nn = neonatal myosin heavy chain isoform. 2b = myosin heavy chain 2b. I = myosin heavy chain I. B) Relative MyHC 2b isoform protein percentages in digastric muscle samples of different age groups. ** indicates *p* ≤ .01. Mean and SEM. *n = 5–7 (p11)*, *n = 7–9 (5–6 weeks)*.

Because the ADG and PDG muscles showed significant reductions of MyHC 2b protein in 5–6 week old Ts65Dn, we next sought to evaluate the developmental trajectory of MyHC 2b expression changes over time in these muscles. MyHC 2b levels were quantified in an 11-day old age group in addition to the 5–6 week old age group shown in Fig 1. Fig 2A shows examples of gels used to determine the relative prevalence of MyHC isoforms in the ADG and PDG muscles of Ts65Dn mice and age-matched euploid controls. For ADG, analysis of MyHC 2b levels by 2-way analysis of variance (ANOVA) revealed significant main effects for both genotype and age in the absence of significant interactions. The ADG of Ts65Dn groups showed significantly less MyHC 2b than the ADG of euploid control groups [F(1,23) = 8.84, p = 0.007], and younger groups showed significantly less MyHC 2b in the ADG as compared older groups [F(1,23) = 89.90, p < .0001]. Analysis of the PDG revealed a main effect for genotype, but not for age, and revealed no significant interactions. Ts65Dn groups showed significantly less MyHC 2b than the PDG of euploid control groups [F(1,24) = 10.41, p = .0036]. Reduction of MyHC 2b percentages in Ts65Dn groups were more striking in the PDG (Ts65Dn: 6.83%, Euploid: 13.79%) than in the ADG (TS65Dn: 19.58%, Euploid: 24.80%) (Fig 2B).

### Histological analysis of digastric muscles

**Fig 3:**
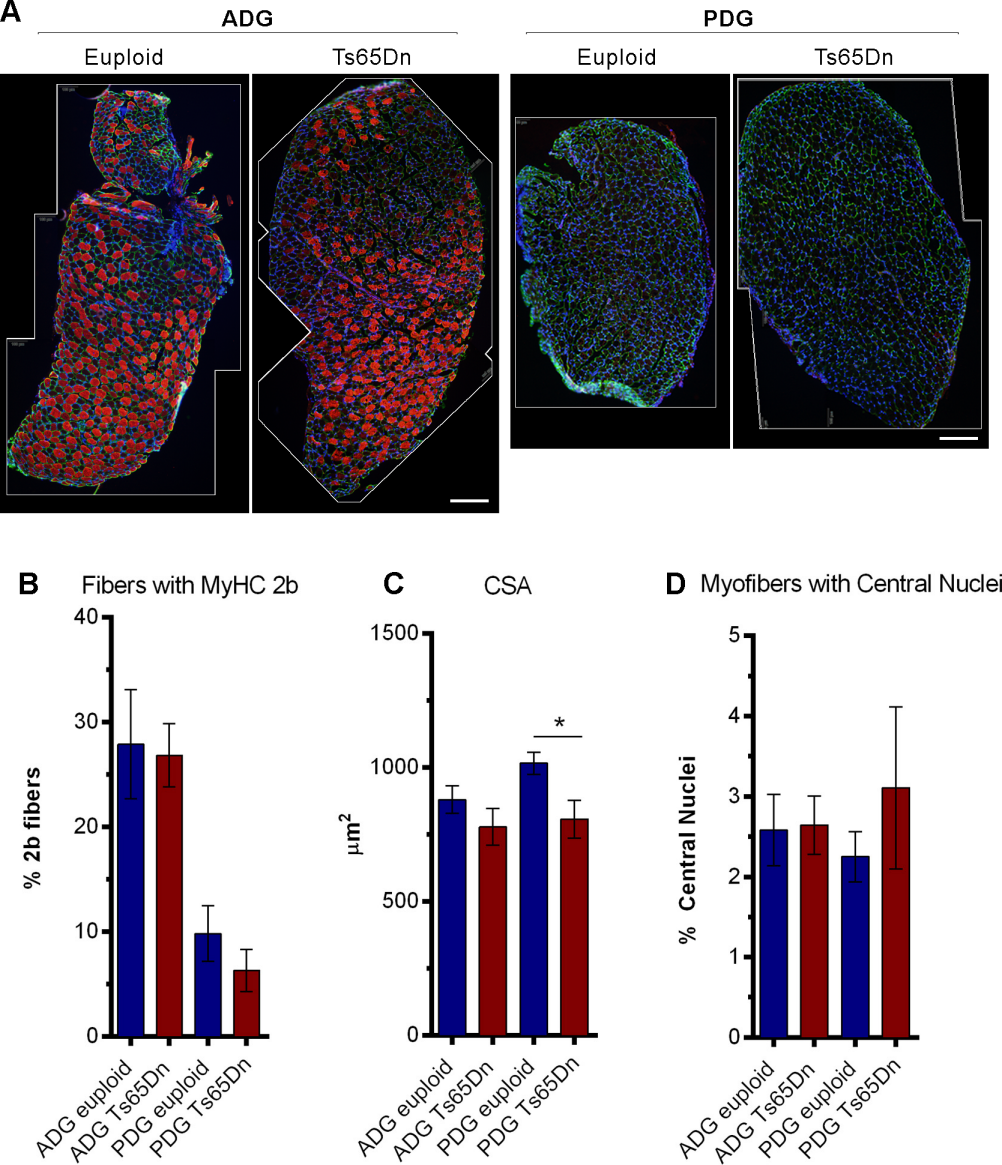
Immunofluorescence staining of thin sections. A) Anterior (ADG) and posterior (PDG) digastric muscle cross sections. Photomontage borders are indicated in white. MyHC 2b (Red), Laminin (Green), Nuclei (Blue). Scale: 200 μm. B) Percentage of myofibers staining positive for MyHC 2b. n = 5–7 per group. Mean and SEM. C) Cross-sectional area of myofibers. n = 4–6 per group. Mean and SEM D) Percentage of myofibers positive for centralized nuclei. n = 6–7 per group. Mean and SEM.

Thin sections (10 μm) of the ADG and PDG muscle in young mice were assessed by immunofluorescence for fibers expressing MyHC 2b, myofiber cross-sectional area, and myonuclei positioning (Fig 3). ADG and PDG cross-sections of Ts65Dn appeared qualitatively normal and similar to euploid in overall appearance and MyHC 2b distribution (Fig 3A). The Ts65Dn group did not show significant quantitative differences in the percentage of myofibers positive for MyHC 2b relative to the euploid control group in either muscle. In the PDG, Ts65Dn myofiber cross-sectional area (CSA) was significantly reduced compared to the euploid group [t(8) = 2.57, p = .03], although CSA differences were not significant in the ADG. Both genotypes showed a mean incidence of myofibers with centralized nuclei that was within the range expected for normal muscle[32].

### Feeding and Swallowing

**Fig 4:**
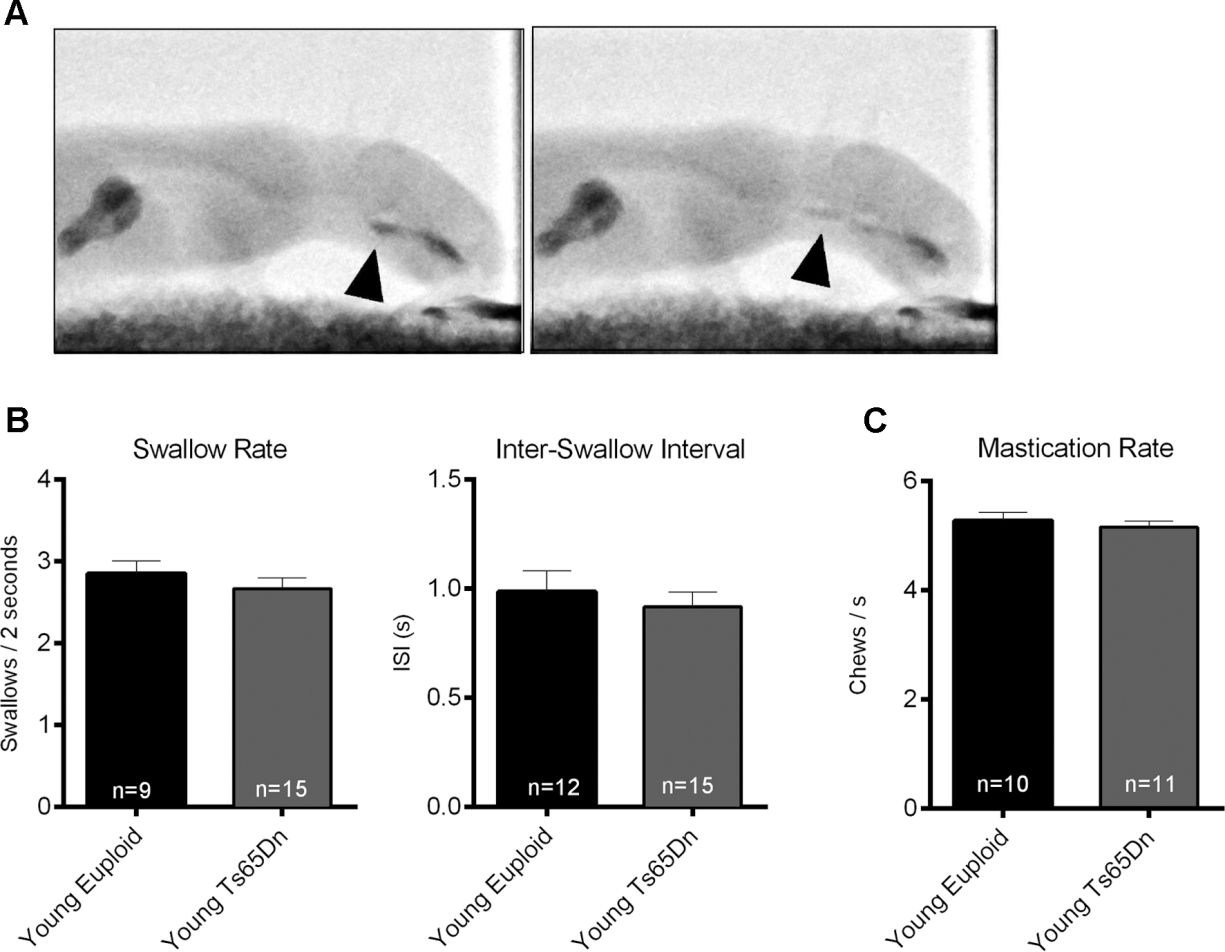
**Behavioral Feeding and Swallowing Assays of Young Mice:** A) Videofluoroscopy swallow study stills with black arrows indicating the food bolus at the valleculae in the frame immediately prior to a swallow (left), and a subsequent frame showing the bolus in the esophagus (right). B) *Left panel*: Videofluoroscopic swallow study (VFSS) analysis of a cheese puree, showing the number of swallows per 2-second intervals. Means and SEM. *Right Panel*: Inter-swallow Intervals, measured in seconds, quantified from the same studies. Means and SEM. C) Mastication rate during feeding on a hard food pellet, assessed by videorecording.

Because the digastric muscles are involved in the movements of the hyolaryngeal complex during swallowing[33], we examined swallowing performance of Ts65Dn and euploid groups (Fig 4A). In assessments of 5–6 week old mice, videofluoroscopic studies quantifying feeding of a cheese puree did not reveal significant differences between genotypes. Compared with the euploid control group, Ts65Dn showed similar swallow rates as determined by the number of swallows during 2-second intervals of continuous feeding. Similarly, inter-swallow intervals (ISI) were of similar duration in the Ts65Dn group as in the euploid control group (Fig 4B). Because the digastric muscles are also involved in opening the jaw during chewing, and increases in MyHC 2b levels of the digastric muscles coincide with developmental transitions to mastication, we assessed rates of mastication in young Ts65Dn and euploid controls. Analysis of mastication rates of mice eating standard hard mouse chow revealed no significant differences between groups (Fig 4C).

### Expression of MyHC Isoform mRNA in digastric muscles of adult Ts65Dn

**Fig 5:**
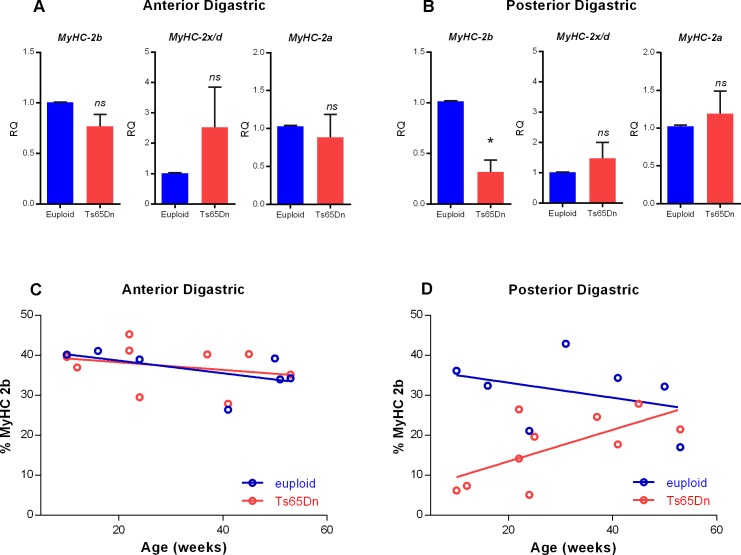
MyHC 2b gene and protein expression in adult mice. qRT-PCR analysis of MyHC isoform expression in A) Anterior Digastric (ADG) muscle, and B) Posterior Digastric (PDG) muscle of 10–11 wk old Ts65Dn as compared to euploid sibling controls. MyHC 2b *(n = 4 per group)*, MyHC 2x/d *(n = 3 per group)*, MyHC 2a *(n = 4 per group)*. * indicates *p* ≤ .05. Bars show mean and SEM. C) Relative amounts of MyHC 2b protein in the ADG of adult mice shown by weeks of age. D) Relative amounts of MyHC 2b protein in the PDG of adult mice shown by weeks of age.

Because ADG and PDG muscles of young mice were found to have reduced levels of MyHC 2b protein as assessed by SDS-PAGE, expression levels of MyHC isoform transcripts were assessed through qRT-PCR analysis of ADG and PDG muscles from 8 adult mice (4 Ts65Dn, 4 euploid), 10–11 weeks old. The euploid control was set as the biological reference sample for each experimental iteration, and statistical significance was assessed though one-sample t-tests. Ts65Dn showed significantly less MyHC 2b transcript in the PDG than the euploid control group [t(3) = 5.760, p = .01]. For both ADG and PDG, the MyHC 2x/d and MyHC 2a isoforms showed no significant differences in expression levels between the two genotypes (Fig 5A and 5B).

### Expression of MyHC Isoform 2b protein in digastric muscles of adult Ts65Dn

We next sought to evaluate the trajectory of MyHC 2b level changes in Ts65Dn as compared to euploid controls in the digastric muscles across an adult age range. Our rationale for performing this analysis was: (1) relative reductions in MyHC 2b levels can occur in cranial muscles as a consequence of aging[28], and (2) the Ts65Dn mouse model has been reported to demonstrate hallmarks of both developmental delay and premature aging[34, 35]. To assess MyHC 2b changes over a maturational time span from early adulthood to old age, data were plotted as age in weeks versus relative percentage of MyHC 2b in the ADG and the PDG (Fig 5C), and significance was assessed by ANOVA. For PDG a significant interaction effect was found for age and the Ts65Dn genotype [F(1,13) = 4.88, p = .05]. Younger adult Ts65Dn showed greater relative reductions in MyHC 2b in the PDG than did older adult Ts65Dn. This is compatible with the conclusion that adult Ts65Dn show age-dependent MyHC isoform profile shifts in the PDG that are different from those of euploid controls. In contrast, no significant effects for age or genotype were found in the ADG of adult mice [F(3,12) = .79, p = .52].

## Discussion

Down syndrome in humans is associated with a high incidence of oral motor impairments, feeding disorders, and dysphagia. Therefore, we hypothesized that the Ts65Dn mouse model of DS would demonstrate biochemical differences in muscles involved in oral movements, as well as behavioral differences in feeding and swallowing tasks. Collectively, the findings of this study support the hypothesis of altered MyHC profiles of some muscles involved in oral movement. Although GG and SG muscles showed unaffected MyHC 2b isoform levels in young Ts65Dn, ADG and PDG muscles of young Ts65Dn differed from euploid control groups in MyHC 2b levels as assessed by SDS-PAGE. However, we did not detect significant differences in measures of feeding movements in young Ts65Dn mice.

The digastric muscles are involved in jaw movement and in typical mice these muscles are known to upregulate MyHC 2b expression during the period of post-natal development that coincides with weaning, or the developmental transition from suckling to chewing and swallowing solid food [19, 20]. Findings of MyHC isoform phenotypes in the ADG and PDG in assays of two age points during post-natal development in Ts65Dn are of interest in light of the developmental nature of some oral motor and feeding difficulties associated with DS. Although MyHC 2b levels were found to be affected in both the ADG and PDG of Ts65Dn, SDS-PAGE analysis of protein suggests that this biochemical phenotype is more striking and severe in the posterior digastric muscle. This finding adds to a small number of prior studies reporting alterations in the MyHC isoform composition of head and neck muscles in mouse models of disorders affecting feeding. In microphthalmic mice that have tooth eruption deficits and receive a powdered diet, levels of MyHC 2b in the digastric muscles were found to be significantly reduced compared with those of WT controls consuming a typical diet, whereas limb muscles in the same mice showed no such differences between groups [36]. Similarly, of the muscles assayed in the current study, results indicated that significant differences in MyHC isoforms in young Ts65Dn were present in the ADG and PDG muscles alone. This result complements a prior study which reported no significant differences in MyHC isoform profiles of soleus muscles of Ts65Dn mice relative to control mice [17]. Thus, it seems possible that in Ts65Dn, there may not be global MyHC isoform phenotypes that affect all muscles, but rather, significant MyHC isoform profile differences may be localized exclusively to certain muscles. If true, this is compatible with the possibility that some oromotor phenotypes that occur in Ts65Dn may be mechanistically distinct from global motor phenotypes that occur in Ts65Dn.

While SDS-PAGE analysis demonstrated lower MyHC 2b expression levels in Ts65Dn digastric muscles than in euploid controls, quantitative analysis of tissue sections stained for the MyHC 2b isoform did not reveal significant differences between genotypes in the percentage of myofibers positive for the MyHC 2b isoform (Fig 3). As has been suggested previously, analysis of MyHC isoform profiles through SDS-PAGE may provide a more sensitive indication of total MyHC isoform levels in a muscle than immunohistochemistry[37]. While immunofluorescence permits identification of fibers that contain a MyHC isoform, it is less amenable to accurate quantification of the amount of the MyHC isoform present, particularly in the case of muscles that contain transitional myofibers expressing multiple isoforms in varying proportions[38]. By contrast, SDS-PAGE analysis of MyHC isoform profiles incorporates relative increases or decreases of other isoforms in determination of the relative proportion of each isoform that is present. Accordingly, methodological differences may have affected consistency of these findings across assays.

The observation of decreased myofiber CSA in the PDG of young (5–6 week old) Ts65Dn compared to euploid controls is of interest in light of prior findings of decreased myofiber CSA in a limb muscle of adult Ts65Dn [34]. Although reduced myofiber CSA in adult Ts65Dn is compatible with a hypothesis of precocious aging, the biological significance of reduced myofiber CSA in young Ts65Dn is somewhat unclear. While young Ts65Dn mice are generally smaller than euploid controls overall[39], relatively rapid increases in myofiber size can occur during this period of postnatal development in mice[40], raising the possibility that reduced CSA in young Ts65Dn may involve a developmental difference.

Despite MyHC isoform phenotypes of the ADG and PDG muscles indicated by SDS-PAGE, videofluoroscopy indicated no significant differences in rates of swallowing or inter-swallow intervals in young Ts65Dn mice as compared to controls during continuous eating of a puree. Young mice also showed no significant differences in mastication rates while eating a hard food pellet. This finding may be of use for on-going efforts to identify an experimental system and measures that recapitulate challenges related to feeding and swallowing that occur with increased frequency in humans with DS, which can include oral and pharyngeal dysphagia, esophageal dysmotility, and reduced tongue pressures^,^[3, 4, 8, 9, 41, 42].

Efficient swallowing involves multiple muscles that can share functional redundancy or compensation in movement of the jaw and hyolaryngeal complex. Thus, in young Ts65Dn mice, functional consequences of atypical muscle biochemistry in the anterior and posterior digastric muscle may be ameliorated by unaffected muscle biochemistry in the other muscles of mastication. The unaffected mastication rates of Ts65Dn in our assays are in contrast to earlier studies of mastication performance in humans with DS. Individuals with DS have shown mastication differences significant for longer durations of chewing, slower chewing, open-mouthed chewing, and food refusal[6, 43]. Therefore, in Ts65Dn, mastication rate as a measure of feeding performance using standard mouse chow may not be as useful as other measures in assessments of oromotor differences associated with DS. In the future, oromotor and swallowing assessments of mouse models of Down syndrome may benefit from recent advances in high-resolution imaging and additional quantitative measures, which can permit detection of relatively subtle perturbations of feeding and swallowing function [31].

Finally, assessment of MyHC 2b levels in aging adult mice (Fig 5C–5D) indicated that striking genotype-specific differences occur in the PDG muscle alone, whereas the ADG appears unaffected. These differences may have future utility for the study of developmental mechanisms of impairment in DS, because of the muscles surveyed, the PDG is derived from the 2^nd^ branchial arch and is innervated by the facial nerve; whereas the ADG has distinct developmental origins and innervation[44]. Our findings of a MyHC isoform protein phenotype in the PDG of Ts65Dn, taken together with reports that DS involves poor gross muscle differentiation specifically of muscles derived from the 2^nd^ branchial arch[45] raise the possibility that the anterior and posterior bellies of the digastric may each be affected in DS through different mechanisms.

In conclusion, assessment of Ts65Dn for phenotypes pertinent to feeding and swallowing revealed biochemical differences of the ADG and PDG muscles in young mice of this model, normal swallowing and mastication rates in behavioral assays of young mice, and biochemical differences of the PDG, but not the ADG, in adult Ts65Dn mice. Further characterization of neuromuscular phenotypes of the head and neck in mouse models of DS may ultimately prove beneficial for translational research efforts to address oromotor and feeding difficulties that are associated with this syndrome.

## Supporting Information

S1 Fig Western Blot of MyHC isoforms. Relative position of MyHC 2b isoform in silver stained gels confirmed by western blot. A) Antibodies specific to MyHC isoforms confirm relative position of bands within limb muscle control samples. 2a = MyHC 2a, 2x = MyHC 2x, 2b = MyHC 2b, I = MyHC I. B) In a silver stained gel, limb muscle control samples indicate the relative positions of bands present in ADG and PDG muscles.
(TIF)

S1 Table qRT-PCR primers used in this study. (DOCX)
