# Supplementary figures and images for: Digastric Muscle Phenotypes of the Ts65Dn Mouse Model of Down Syndrome
Source: PLoS One. 2016 Jun 23;11(6):e0158008. doi: 10.1371/journal.pone.0158008 (PMC4919106; doi:10.1371/journal.pone.0158008)

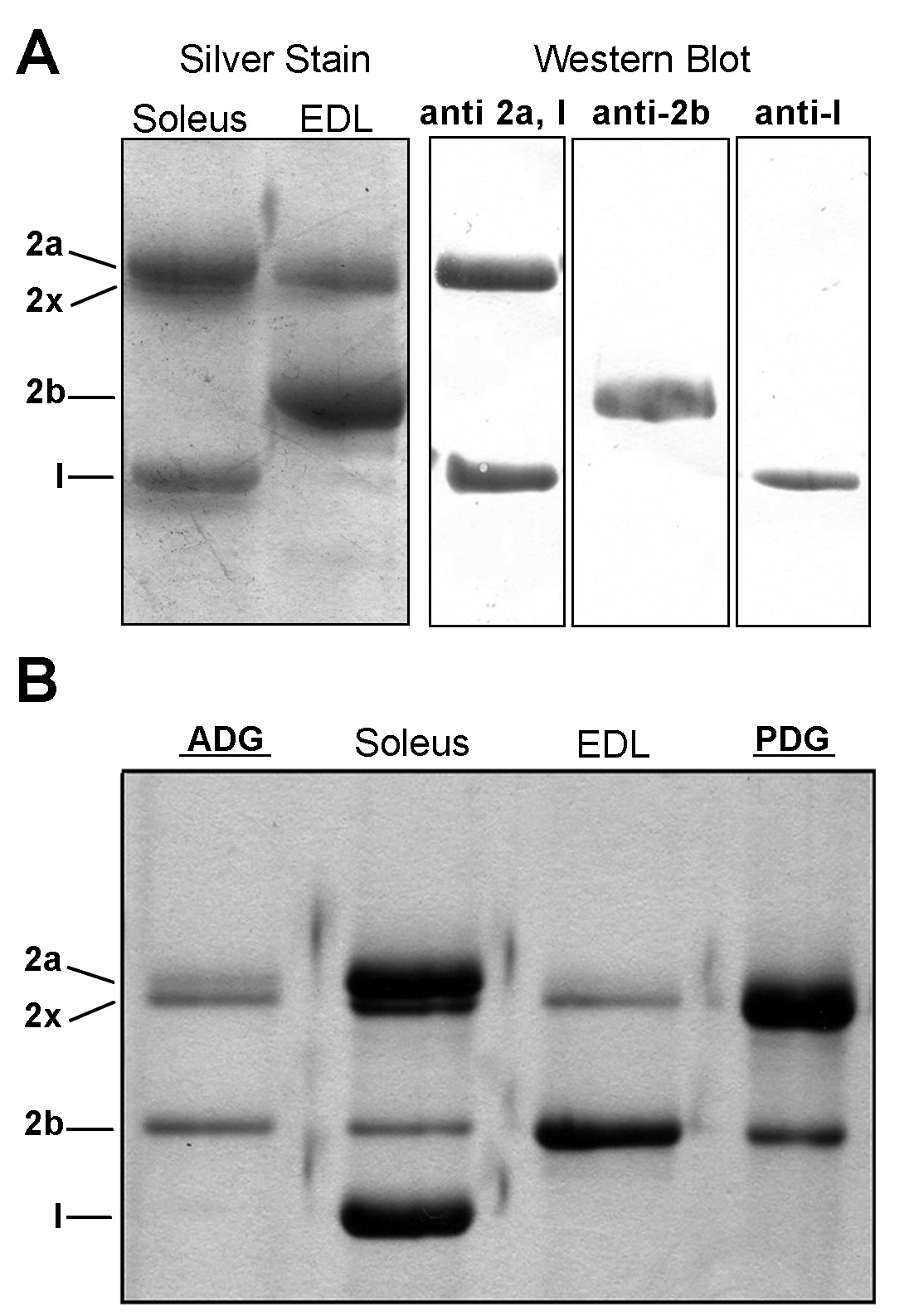

Supplement: S1 Fig — Relative position of MyHC 2b isoform in silver stained gels confirmed by western blot. A) Antibodies specific to MyHC isoforms confirm relative position of bands within limb muscle control samples. 2a = MyHC 2a, 2x = MyHC 2x, 2b = MyHC 2b, I = MyHC I. B) In a silver stained gel, limb muscle control samples indicate the relative positions of bands present in ADG and PDG muscles. (TIF) [file pone.0158008.s001.tif]
