# Supplementary material for: Digastric Muscle Phenotypes of the Ts65Dn Mouse Model of Down Syndrome
Source: PLoS One. 2016 Jun 23;11(6):e0158008. doi: 10.1371/journal.pone.0158008 (PMC4919106; doi:10.1371/journal.pone.0158008)
Supplement: S1 Table — (DOCX) [file pone.0158008.s002.docx]

**S1 Table: qRT-PCR primers**

| **Isoform / Gene Name** | **Taqman Assay ID** |
| --- | --- |
| MyHC-2a / *myh2* | Mm00454982_m1 |
| MyHC-2x/d / *myh1* | Mm01332489_m1 |
| MyHC-2b / *myh4* | Mm01332518_m1 |
| *Gapdh* | Mm99999915_g1 |
| *B-actin* | Mm00607939_s1 |
